# Ionic Actuators as Manipulators for Microscopy

**DOI:** 10.3389/frobt.2019.00140

**Published:** 2019-12-20

**Authors:** Indrek Must, Pille Rinne, Friedrich Krull, Friedrich Kaasik, Urmas Johanson, Alvo Aabloo

**Affiliations:** Intelligent Materials and Systems Lab, Institute of Technology, University of Tartu, Tartu, Estonia

**Keywords:** manipulators, ionic and capacitive laminates, ionic liquids, vacuum, compliant actuation, SEM

## Abstract

Non-destructive handling of soft biological samples at the cellular level is becoming increasingly relevant in life sciences. In particular, spatially dense arrangements of soft manipulators with the capability of *in situ* monitoring via optical and electron microscopes promises new and exciting experimental techniques. The currently available manipulation technologies offer high positioning accuracy, yet these devices significantly grow in complexity in achieving compliance. We explore soft and compliant actuator material with a mechanical response similar to gel-like samples for perspective miniaturized manipulators. First, we demonstrate three techniques for rendering the bulk sheet-like electroactive material, the ionic and capacitive laminate (ICL), into a practical manipulator. We then show that these manipulators are also highly compatible with electron optics. Finally, we explore the performance of an ICL manipulator in handling a single large cell. Intrinsic compliance, miniature size, simple current-driven actuation, and negligible interference with the imaging technologies suggest a considerable perspective for the ICL in spatially dense arrays of compliant manipulators for microscopy.

## Introduction

Precise biomedical applications often involve manipulation of soft and fragile objects: gel-like substances or delicate samples of biological origin. Biological systems on any scale (from viruses and cells to organs) create mechanical cues in response to mechanical forces being applied to them (Krieg et al., 2019), implying immense importance of scalable and compliant manipulation techniques for mechanobiology studies. Highly repeatable manipulators are commercially available at nanometer-scale positioning accuracy, rendering closed-loop systems that do not require human intervention for positioning, such as the atomic force microscope (AFM), the leading platform for mechanobiological research (Castillo et al., 2009; Alsteens et al., 2017). In addition to piezoelectric actuators, as in AFM instruments, thermoelectric and electrostatic actuators prevail among the miniaturized contact-based manipulators. Actuators linked to microelectromechanical systems (MEMS) enable cellular- and subcellular-level manipulation (Verotti et al., 2017).

Compliance is one of the essential characteristics of both hard and soft robotic systems. Conventional (industrial) hard robots made of rigid materials (e.g., metal) can be engineered to be compliant toward softer biological subjects (such as humans) mainly thanks to their highly sensitive sensors and high-precision control algorithms. However, in case of grave malfunction, these systems could cause serious damage, creating the need for intrinsically soft devices (Polygerinos et al., 2017). In soft robotics, the compliance of the robot (e.g., a manipulator) is sometimes achieved by matching Young's moduli of its constituent materials (Majidi, 2014; Rus and Tolley, 2015; Coyle et al., 2018). Moreover, soft robotic systems bioinspired by vertebrates, where the properties of constituent materials span several magnitudes of moduli (from soft tissue to bone) (Yang et al., 2018) can still be considered compliant thanks to the seamless integration.

The soft (biological) samples are often widely distributed cellular structures with their characteristic dimensions and mechanical properties varying widely between specimens and in time. Consequently, scalable and compliant manipulators are favored for biomaterial manipulation, whereas the absolute positional accuracy can be of secondary importance. Moreover, if a large number of semi-independent actuators are involved, then the feedback-loop-based systems (as in AFM instruments) start to increase in complexity exponentially. Furthermore, in some cases, larger biological systems (above 100 μm) than just a single cell need to be characterized (e.g., when the mechanical properties also depend on the interactions with the extracellular matrix). In such cases, much larger probes than the standard AFM probe are needed for accurate characterization (Andolfi et al., 2019). The intrinsic compliance, miniaturizaibility, and excellent scalability of soft manipulators enabled by shape-morphing materials can enable new *in situ* methods for cellular-level manipulation (Jager et al., 2000b). Electromechanically active actuators have been demonstrated in scale relevant to microscopy studies (Jager et al., 2000a). However, rendering the morphing materials into practical manipulators still poses numerous challenges—in particular, concerning the useful/relevant device configurations, their cycling stability in various operating environments, and their possible interference with the *in situ* visualization techniques.

We explore the application of gel-like actuators in the manipulation of soft samples. As biological (gel-like) samples commonly exhibit viscoelastic properties (Muñoz and Albo, 2013), the viscoelasticity can be considered relevant for the living organisms in physical interactions with the environment. Therefore, also manipulators that exhibit viscoelastic properties also could be beneficial for enhanced contact with soft samples. Instead of using an additional viscoelastic joint between an actuator and the contact point to the sample, we propose actuators that are viscoelastic thanks to their mobile liquid phase component. Liquid-containing compliant manipulators are known to minimize sample damage and enable advanced physical interaction modes specific to soft bodies (Polygerinos et al., 2017). Indeed, also soft fluid-containing electroactive actuators demonstrate intrinsic viscoelasticity (Vunder et al., 2012). Such electroactive laminate achieves the advantages of fluidic actuators on a scale that is already challenging to fabricate using soft lithography.

**Figure 1:**
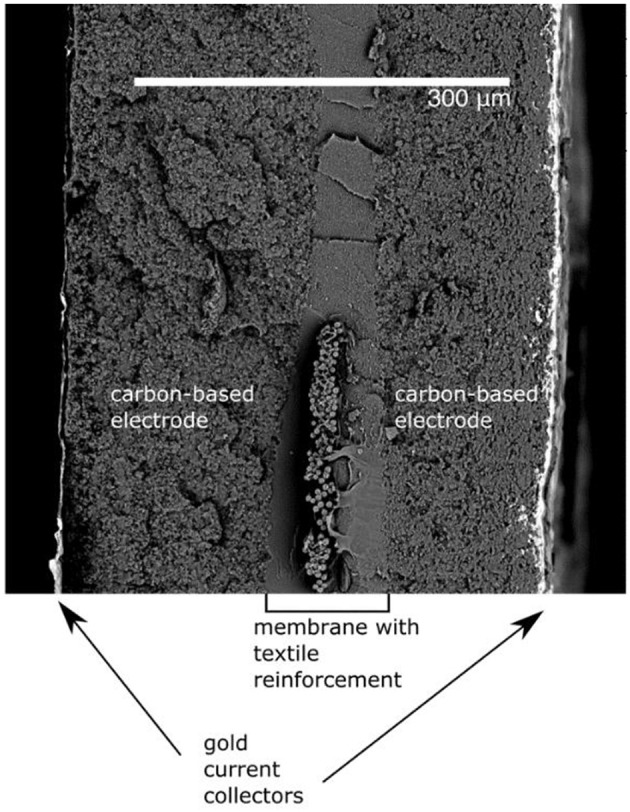
Cryofractured cross-section of ICL.

We demonstrate μm- to mm-scale manipulators based on ionic and capacitive laminates (ICLs). ICLs have an exclusive set of desirable properties for this purpose. Their soft and compliant nature has made ICL materials appealing to various biomimetic soft robotics applications (Must et al., 2015; Hines et al., 2017). ICL is composed of two polarizable electrodes separated by an ion-conductive open-pore network gel layer. Upon applying a potential difference between the electrodes, the anions and cations in the liquid phase of the gel are electrostatically attracted to the positively and negatively polarized electrodes, respectively. As a consequence of this charging, the ions of the electrolyte (e.g., ionic liquid) inside the gel get displaced toward the respectively polarized electrodes. Since the anions and cations of the electrolyte differ in size, mobility, and physicochemical properties, a swelling ratio imbalance results in the composite. This, in turn, causes the laminar actuator to bend toward the positively polarized electrode typically. See Figure 1 for a detailed description of the actuator structure.

The mobile electrified liquid phase incorporated within the gel network gives the actuator a viscous response in addition to the elastic component. In the context of manipulation, the resulting viscoelastic property is beneficial: the elastic response of the actuator promises a good match for *in situ* manipulation under optical as well as electron microscopes while the viscous response contributes to compliance and provides an intrinsic cutout of high frequencies for noise cancellation. Importantly, the electrostatically driven non-electrochemical processes in ICLs have shown to result in very high cycling stability and lifetime (Kaasik et al., 2017). Contrary to solvent-based electrolytes, ionic liquids as ICL electrolytes and gel-swelling agents allow operation of the actuators in the air as well as in a vacuum, thanks to the negligible vapor pressure of ionic liquids. Low-voltage non-magnetic and non-thermal activation excludes interference with the electron optics of scanning electron microscopes, also enabling *in situ* SEM observation. Despite the listed favorable properties of the basic ICL material, rendering the laminate into practical manipulators still poses several challenges that are addressed here by suggesting possible configurations in relevant case studies.

## Materials and Methods

### Fabrication of ICL Actuators

The ICL material is a three-layer composite with an ion-conductive membrane sandwiched between two electron-conductive electrodes—a structure that is similar to the electrical double-layer capacitors. The material used for the preparation of manipulators in this study was fabricated using an industrially scalable ICL manufacturing method that has been previously described in detail elsewhere (Kaasik et al., 2017). In short, fine textile with inert fibers [e.g., silk fabric (11.5 g/m^2^, Esaki) or glass fiber fabric (18 g/m^2^, Storm RC World)] was fastened and tautened onto a frame. Then the membrane solution was applied to the fabric using a brush. After the evaporation of solvents, an open-pore ion-conductive polymeric gel membrane was formed. Then, electrodes were sprayed on both sides of the membrane using an airbrush (Iwata). Finally, gold leaf (Giusto Manetti Battiloro 24K transfer leaves) current collectors were glued on the laminate using a rolling method similar to what has been previously described elsewhere (Must et al., 2015). The final thickness of the composite is very well tunable. In the comparative performance study introduced in section Tool Attachment—Bending Stiffness Matching, a 150–160 μm thick laminate was used. The actuator material was prepared at room temperature and at a relative humidity between 65 and 75%.

The cross-section of the structure of the prepared material is presented in Figure 1. The thickness of the imaged actuator exceeds that of actuators prepared for manipulation. Therefore, all the layers are visible from the cryofractured cross-section.

The prepared bulk material can be cut into various shapes to obtain functional manipulators. However, this basic manufacturing procedure can also be modified to integrate various probes and sensors directly into the active material. In section Manipulator Concepts and Their Fabrication, we will describe four manipulator concepts, from the simplest to more advanced ones, that are all based on the same ICL material described above. As the four concepts share most of the manufacturing steps, the corresponding descriptions in sections below cover only the necessary modifications to the main ICL preparation procedure to obtain the more advanced manipulator.

### Manipulator Concepts and Their Fabrication

#### A Cantilevered Actuator Directly as a Manipulator

**Figure 2:**
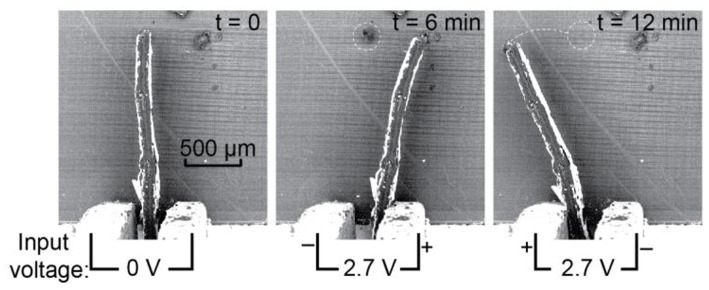
*In situ* scanning electron micrographs of a miniaturized ICL actuator used directly as a manipulator; displacement of small objects on the SEM stage surface is demonstrated.

In the simplest embodiment, the ICL can be cut into a cantilever and used to apply force to an object via direct contact with the actuator's side or the electrode area. This configuration can be useful in applications where manipulators are needed for extremely confined spaces. Since the ICL thickness is controllable in the manufacturing process, and its other dimensions are easily miniaturizable in the cutting and shaping stages; therefore the final dimensions of the actuator/manipulator could even span less than a few cubic millimeters per manipulator. Figure 2 shows the *in situ* scanning electron micrographs of an ICL actuator moving objects on the SEM stage surface.

#### Active Tweezers

**Figure 3:**
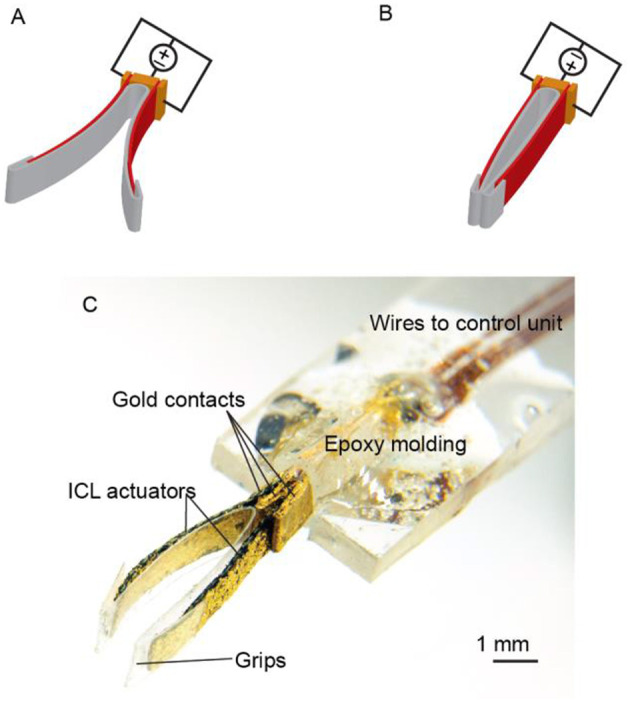
Electroactive tweezers based on ICL actuators. **(A,B)** Tweezers in the open and closed positions, respectively. **(C)** Photograph of the final device.

In a more complicated embodiment, two cantilevered ICL actuators can be stacked together and operated in opposite phases, forming soft grippers. The conceptual drawing in the open and closed configuration is given in Figures 3A,B, respectively. Typically, this type of actuator material bends toward the positive terminal (although deviations to this general rule exist). Therefore, when positive conventional current is applied to the inward-facing electrodes in respect to the outward-facing electrodes, the distance between the tips of the actuators gets shorter, upon reversing the potential or current, the distance between the tips increases. Alternatively, a longer ICL actuator could also be clamped in the middle to achieve similar gripping-type behavior.

The gripper used in this study was fabricated as described in section Fabrication of ICL Actuators (silk as the base material) with the following modifications. Two 5.0 × 1.2 × 0.15 mm^3^ (length × width × thickness) pieces of ICL (in red) were cut and clamped face-to-face between three gold contacts (in orange), as shown in Figures 3A,B. The inward-facing electrodes, as well as the outward-facing electrodes of the ICL actuators, were commutated together. The gripper arms were lined with a single piece of 30-μm polypropylene foil (in gray) to minimize cross-contamination between the ICL and possibly liquid-containing samples, and to define the contact area better. The final gripper assembly is depicted in Figure 3C.

#### An Active Probe

**Figure 4:**
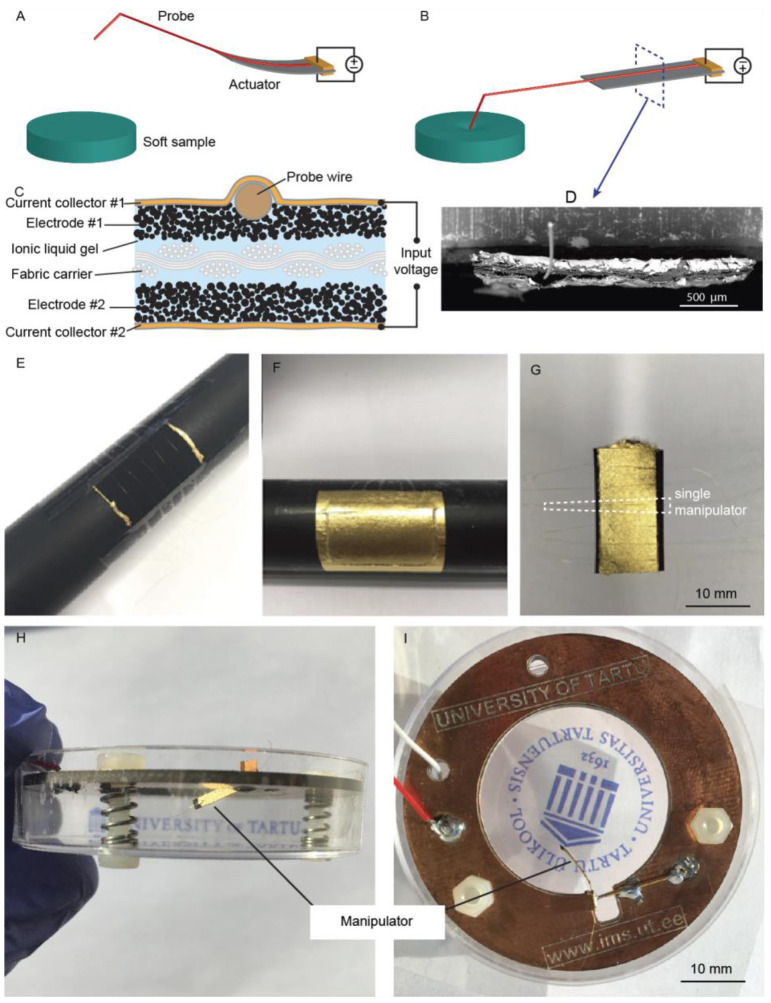
The electroactive probe manipulator concept, shown in **(A)** not contacted and **(B)** contacted mode. **(C)** Schematic and **(D)** SEM image of the cross-section of the ICL manipulator, showing the location of the wire probe. Snapshots recalled from the fabrication: **(E)** metal wires were placed on the electrode surface; **(F)** metal wires were fixed in place under the gold current collector; and, finally, **(G)** the manipulators were formed by cutting. Photographs of the **(H)** side and **(I)** top view the final device.

The next case study considers an ICL actuator with a flexible and conductive probe integrated into the material already during fabrication. For example, various probes and tubes can be integrated into the material in the final step during the current collector attachment. We designed an ICL with an integral wire probe that bends along with the actuator. This can be considered a two-in-one manipulator configuration, where the free end of the cantilevered probe can manipulate a sample mechanically to measure its mechanical properties, and simultaneously, various integrated sensors could be used to evaluate the electrical, electrochemical, and chemical properties of the same sample. Figures 4A,B show the manipulator concept, depicted in the not contacted and contacted state, respectively.

The main challenge with this approach is to select and attach such a probe that a sufficient deflection magnitude is still maintained in the ICL actuator after the integration. The probe's mechanical properties should not interfere with the actuation, whereas it should still be functional as a probe (e.g., sufficiently conductive for conductivity measurements). We chose to integrate a very fine gold-coated tungsten wire (diameter: 30 μm), as illustrated in Figures 4C,D.

The fabrication of this manipulator proceeded as described in section Fabrication of ICL Actuatorswith silk as the supporting structure and with the following modifications. In the current collector attachment step, a 2 × 5 cm piece of the composite was fastened on a metal rod (*d* = 3 cm) using tape, and the first current collector was attached to one side of the laminate as usual. Then, the actuator was removed from the rod and turned over. About 6 cm long gold-coated tungsten wires (diameter: 30 μm) were cut and tightly fastened over the composite on the rod using tape (Figure 4E). After that, the current collector was attached on the top of the wires as usual (Figure 4F). After removing the composite from the rod, it was carefully cut into smaller samples using a scalpel and a metallic ruler aligned with the probe wires (Figure 4G).

The microscope accessory was completed by attaching the manipulator to a custom holder inside a plastic Petri dish that facilitated the probing of liquid samples, as shown in Figures 4H,I. A suitable contact pressure to the actuator was maintained using a spring-loaded pin. The tip of the probe wire was finally shaped (e.g., bent), as needed. For conductivity measurements, a counter electrode was fixed on the bottom of the dish, and the sample in question was placed on top of it.

#### An Active Pipette, Also Functioning as a Tool-Positioner

**Figure 5:**
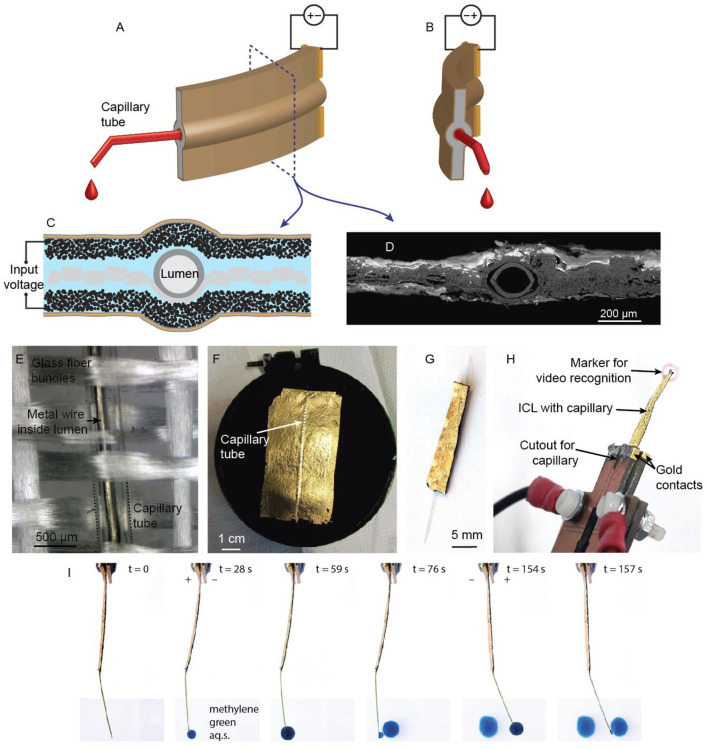
**(A,B)** the concept of an active pipette for positioning fluids in oppositely polarized states. **(C)** The cross-section diagram and **(D)** an SEM micrograph of the mosquito proboscis-inspired ICL-pipette assembly, showing the arrangement of the capillary tube in the center of the laminate. **(E)** A macro photograph of the glass fiber cloth with an interwoven capillary tube before application of the membrane. **(F)** The active pipette after application of electroactive layers; the elevated strip indicates the location of the capillary tube. **(G)** The finished active pipette. **(H)** The active pipette in its holder, showing the gold contacts and a marker attached for video recognition. **(I)** Deposition of methylene green dye by an electrically steerable ICL-pipette.

The most involved approach for preparing ICL-probe assemblies is integrating the probe (e.g., wire or tubing) already in the early stages of the composite preparation. For example, we integrated inert, flexible non-conductive PTFE, PET, or polyimide tubes into the membrane of the actuator. Inspired by the proboscis of a mosquito, the ICL-tube assembly can behave as an active pipette tip for depositing or extracting liquids to/from desired areas, as schematically shown in Figures 5A,B, yet preserving compliance to soft tissues. Moreover, if some of the supporting fibers of the ICL membrane have been replaced with tubes, these now hollow fibers could be used for inserting other probes or sensors (e.g., the same conductivity probe introduced in the previous section) into the actuator. This would enable fast and easy switching between tools or sensors during an experiment.

The fabrication of an active capillary manipulator started with the integration of a capillary tube into the supporting fabric structure to achieve the centermost position for the tube. One fiber bundle was first removed from the glass fabric and then replaced with a tube (e.g., PTFE: 254 μm inner diameter, 76 μm wall thickness; polyimide: 127 μm inner diameter, 20 μm wall thickness; or PET: 300 μm inner diameter, 3.8 μm wall thickness) (Figure 5C). The fabrication then proceeded, as described in section Fabrication of ICL Actuators. The scanning electron micrograph in Figure 5D confirms the central location of the tube. Figure 5E shows the micrograph of the capillary tube woven in-between the glass fiber bundles. A metallic wire has been inserted into the tube for better visualization. Figure 5F gives a snapshot of the fabrication process after the attachment of the gold current collector, showing the elevated surface feature that reveals the location of the capillary tube. Figure 5G shows the finished active pipette tip. The final assembly was attached to gold electrical terminals from its base and equipped with a visually distinguishable marker at its tip for displacement detection via video recognition, as shown in Figure 5H. Figure 5I and Supplementary Video 1 show an active pipette with PI tubing dispensing droplets of aqueous methylene green solution into different locations on a microscope slide.

### Microscopy

Hitachi TM3000 scanning electron microscope was used at 15 keV acceleration voltage to visualize the probing of soft samples in vacuum. The actuator was fastened between gold clamps commutated to a DC power supply.

The cross-sections of actuators were visualized with the same Hitachi TM3000 scanning electron microscope. Samples for SEM micrographs were first cryofractured using liquid nitrogen to obtain clearer cross-sections and then taped on a metal sample holder with their cross-sections facing up. The samples, together with the sample holder, were sputtered with 5 nm of gold using a Leica EM ACE600 sputter coater to obtain more detailed SEM micrographs.

5x and 10x objectives were used for optical microscopy.

### Displacement Characterization

The actuation of active tweezers was characterized by post-processing the recorded videos with video recognition implemented in the Vision Development module in LabView, National Instruments. Digital templates were created of visually distinctive areas (edge and surface defects from intentionally rough cutting, or visual markers attached for the detection purpose) and tracked frame-by-frame using the grayscale value pyramid pattern-matching algorithm.

**Figure 6:**
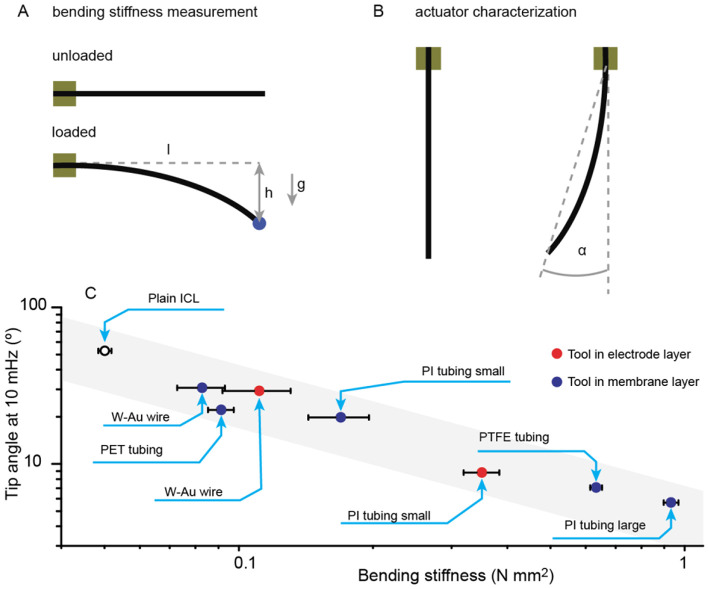
**(A)** bending stiffness measurement setup; **(B)** bending angle definition; **(C)** Manipulator deflection with ±2 V 10 mHz triangular input signal for ICLs carrying denoted tools vs. their bending stiffness.

The influence of various integrated probes was comparatively studied on samples from the same batch with different integrated probes (Figure 6C). The displacement detection method for this comparative study was done as previously described in detail elsewhere (Punning et al., 2014). In brief, the actuator's (free length: 18 mm) displacement was video recorded, and the actuator's tip angle, defined in Figure 6B, was extracted by post-processing in LabView.

### Electrical Characterization

Electrical driving signals were generated, and impedances registered using IVIUM CompactStat and Princeton Applied Research Parstat 2273 electrochemical workstations. All electrical measurements were done using electrochemically inactive gold contacts.

The measurement setup for the comparative performance study (Figure 6B) has been previously described elsewhere in detail (Punning et al., 2014). In the comparative study, we used triangular voltage waves (cyclic voltammetry) to drive the actuators. The electrochemical aspects of the material and its capacitive nature have been described in detail elsewhere (Kaasik et al., 2017).

### Mechanical Characterization

The mechanical characterization of non-homogeneous multilayer composite materials with integrated probes is not straightforward. For example, tensile testing in the same direction as the reinforcement fibers is expected to be dominated mostly by the mechanical properties of the fibers (characteristic values in the GPa range) and not by the bulk active material itself (characteristic values in the MPa range). Since bending performance is the most important characteristic of this type of actuators, it is most reasonable to use bending stiffness (the actuator's resistance to bending deformation) to characterize and compare different manipulator configurations. Bending stiffness is a function of Young's modulus and the area moment of inertia of the sample.

The bending stiffness of the bare ICL material and the ICL-probe assemblies was characterized using the end-loaded cantilever beam configuration. The actuators were fastened between clamps, and different weights were attached on the tip of the cantilever. The resulting deflection was measured from the video. See Figure 6A for a description of the measurement setup.

The bending stiffness (**BS**) of different manipulator assemblies was calculated using the following formula for end-loaded cantilever beams:

$$\begin{array}{l} BS=\frac{m\times g\times l^{3}}{3\times h}, \end{array}$$

where *m* is the mass of the added load (in blue in Figure 6A; 7.4 and 50.9 mg loads were used), *g* is the gravitational acceleration (9.8 m s^−2^), *l* is the free length of the cantilever beam (18 mm), and *h* is the measured deflection in the cantilever beam due to the added load.

### Samples for Probing

Capelin eggs for manipulation experiments were purchased from the local supermarket. In the conductive probing experiment, a drop of 1-ethyl-3-methylimidazolium trifluoromethanesulfonate ([EMIM][OTf]) ionic liquid was used.

## Results and Discussion

The considered manipulator concepts suggest pathways to transfer the ICL actuators from simple configurations suitable for material characterization toward practical arrangements as microscope accessories. Modifications in various stages of the ICL fabrication enable us to manufacture considerably different functional manipulators. Both electronically conductive (i.e., the metal wire), and non-conductive and inert (i.e., the capillary tube) tools can be integrated into the ICL electrode or membrane during the ICL manufacturing.

### Tool Attachment—Bending Stiffness Matching

In the presented active manipulator concepts, the ICL was used to bend a supplementary component; thus, matching the ICL actuator bending stiffness to its supplement is essential for effective operation. In general, the supplementary components increase the bending stiffness of the manipulator, effectively creating a load for the actuator. Figure 6C compares the actuation performance of ICL manipulators carrying various tools. Indeed, the ICL without a tool attached yielded the largest bending angle; and the general trend shows a linear decrease of actuation magnitude in the log-log-scale. Consequently, it is possible to estimate the bending magnitude of the manipulator by knowing the bending stiffness of the actuator and the tool.

In general, the position of the textile base material determines the location of the ICL's neutral layer, which is the layer with zero strain on bending. However, ICL manipulators were manufactured with tools placed in two locations: in the membrane layer in the center of the laminate, and close to one side of the laminate. In the former case, the tool location coincides with the textile reinforcement; thus, the bending stiffnesses of the tool and the ICL are expected to be additive. Indeed, Figure 6C shows most of the manipulators with tools placed within the membrane following a uniform linear trend. Minor deviations from it could be caused by the part of the electrode deposited on top of the non-conductive and inert tubing that renders this part of the composite electromechanically inactive due to blocked ion flux. Indeed, Figure 6C shows that the manipulator carrying the extremely soft but wide and flat PET tubing yielded actuation performance lower than expected due to a significant decrease in the actuator's effective surface area. Slightly better performance is even achieved with a gold-coated tungsten wire probe integrated into the actuator, although the mechanical properties of metal wire are orders of magnitude stiffer.

The placement of the tool on the surface of the laminate potentially affects the bending performance due to a shift in the location of the neutral layer toward the electrode carrying the tool. Therefore, for more predictable actuation behavior in both directions, it is advisable to attach probes already in the neutral layer (i.e., in the textile). However, from the manufacturing point of view, the ease of attaching probes only in the final manufacturing stage might be a more significant advantage in some cases.

### Driving Considerations, Performance, and Stability

The ICL actuator is a capacitive device very similar to electrical double-layer capacitors (EDLC). The defining difference is that the swelling and contraction of electrodes upon the application of an electric signal that drives the bending motion in ICLs is seen as a disadvantage in EDLC-s. The actuation in the ICL is proportional to the charge stored in it (Kaasik et al., 2017). Indeed, in a capacitive system, input current directly determines the actuation speed, whereas the charge (i.e., the integral of current) determines the distance traveled if all other parameters are kept unchanged. Very typically, the three-layer bending type actuators have previously been controlled using voltage steps, sine or triangular waves in the two-electrode configuration. In the following sections, we will explore the capability of efficiently using current as the process parameter, as small current levels can generally be controlled more precisely than variations in large voltage levels (e.g., microvolt-level voltage variations in an ICL charged to the open-circuit potential of hundreds of mV). Therefore, charge-driven energy-storing actuators, such as the ICLs, prevail in applications that require slow and uniform actuation speeds. However, the electrochemical stability windows of components used in the ICL preparation (i.e., material parameter) as well as the internal resistance of the ICL manipulator (i.e., device parameter) need to be considered in the current-driven mode.

**Figure 7:**
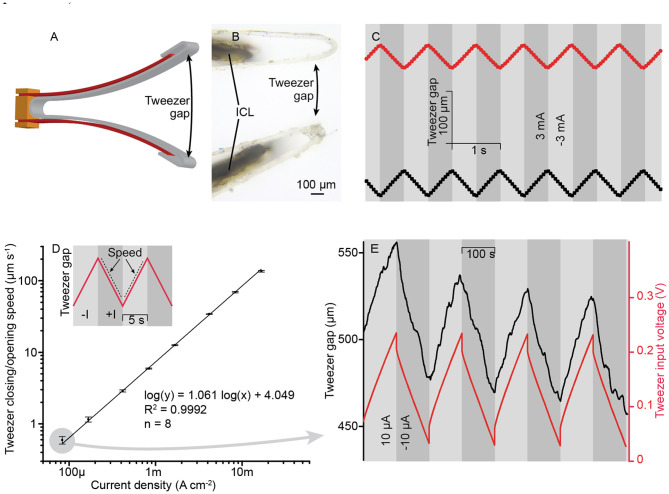
**(A)** Tweezer gap used as the performance metric for the electroactive tweezers. **(B)** Microscope image of the tweezer arm tips showing the grips as contact points. **(C)** The position of tweezer arm tips upon applying a 1-Hz square wave at ±3 mA amplitude. **(D)** The tweezer closing/opening speed in an unobstructed mode at a range of current densities. **(E)** The typical response curve of the electroactive tweezers upon applying an extremely small current, ±10 μA.

Precision control of the ICL using current signals is demonstrated using the tweezer configuration. The ICL tweezer gap, as shown in Figure 7A (microscope image in Figure 7B) formed at the distance of 5.1 mm from the tweezer's base and varied from zero (tweezers closed) to ~2 mm in the experiments, demonstrating a practically sufficient shape variation in the miniaturized scale. Figure 7C shows the active tweezer gap modulation upon applying a 1-Hz square-wave current of 3 mA. The tweezer gap followed an almost ideal triangular pattern, following the course of the charge stored in the capacitive device. The peak-to-peak amplitude of actuation was 86 ± 17 μm (*n* = 90). Figure 7D shows that current levels varied over more than two orders of magnitude during actuation and resulted in a well-defined, close-to-proportional control of the ICL manipulator speed. For practical considerations, the behavior of the manipulator at low current levels (i.e., low speed) is particularly noteworthy. Figure 7E demonstrates the control of ICL tweezers at a rate of only 590 nm s^−1^ by applying a 10-μA input current; the ICL terminal voltage spanned by 0.21 V only. The superimposed drift of the tweezer gap in Figure 7E is characteristic of soft manipulators with a viscous component and can be considered as an advantage for compliantly manipulating soft samples (see also section Compliant Manipulation of Soft Objects). Moreover, this drift (55 nm s^−1^) was much smaller than that imposed by the current actuation signal. Consequently, the ICL manipulator matches well with the typical *in situ* microscopy methods, offering actuation control at fast as well as at slow rates.

**Figure 8:**
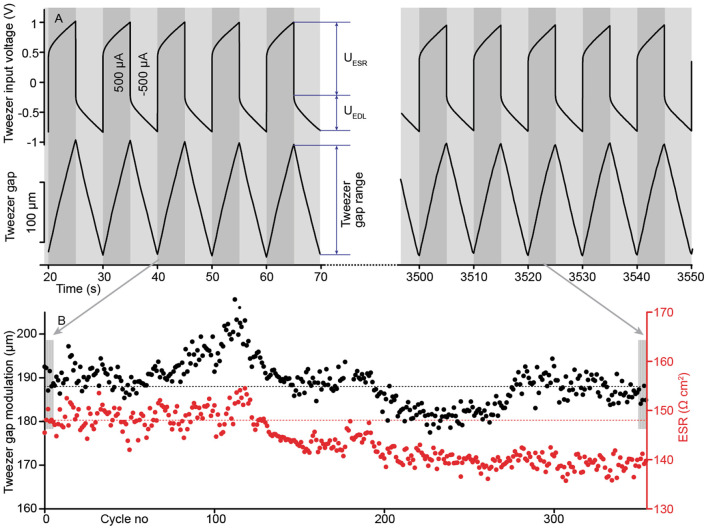
**(A)** Repeatable pattern of the tweezer gap and the corresponding transient voltage, showing the contribution of the equivalent series resistance (ESR). **(B)** Tweezer lifetime and ESR during 350 actuation cycles.

The excellent stability of ICL tweezers is further demonstrated in Figures 8A,B. In 350 cycles at 0.1 Hz rectangular current of 500 μA, the tweezer gap was modulated by 188 ± 15 μm. No pronounced pattern that could be attributed to the degradation of the material was observed (the dotted line represents a guide for the eye).

Further advantages of using current to control the speed of the manipulator are highlighted in Figure 8A. The tweezer's terminal voltage consists of the voltage drop at the equivalent series resistance (ESR), denoted as U_ESR_, and the voltage drop at the electric double-layer at the electrodes denoted as U_EDL_. In the particular current density value, U_ESR_ was larger than U_EDL_. Moreover, the ESR value decreased noticeably (5.5%) during cycling, possibly due to water absorption by the ionic liquid electrolyte and by Joule heating. However, using current as the feedback parameter ensured a uniform triangular actuation pattern throughout the lifetime experiment, deterring possible dependence on the ESR variations and the current density level.

### Compliant Manipulation of Soft Objects

**Figure 9:**
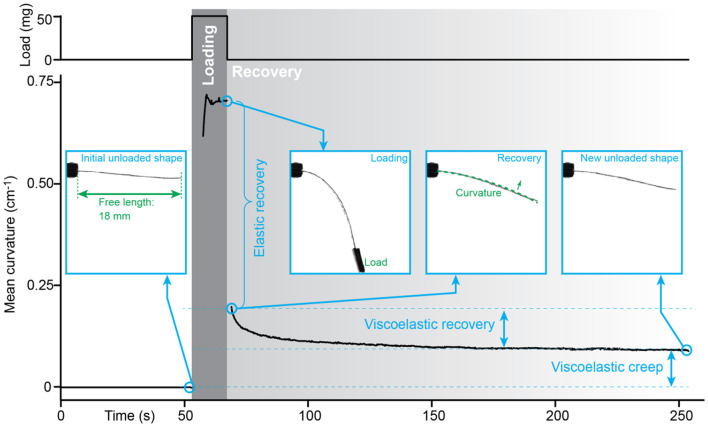
Viscoelastic component in an ICL laminate, evidenced by the inelastic recovery of an ICL post-loading.

The viscoelastic properties of the ICL material imply that the actuator's shape at zero position, i.e., the position with zero charge injected, is tunable and that it also adjusts further according to the applied load. Figure 9 demonstrates the recovery behavior of the ICL laminate in response to applying and removing a load in the gravitational field. As typical for viscoelastic materials that exhibit both elastic and viscous properties, the removal of the load first results in quick elastic recovery that is followed by a slower recovery at a continuously decreasing rate. Depending on the applied load, the viscous recovery might result in permanent deformation that can be useful for preshaping the material into, for example, custom gripper arms. Moreover, this behavior is very beneficial in case of compliant handling of soft samples; the manipulator remains operational after contact thanks to the elastic deformation component, but is safe to the object thanks to the viscous deformation component. The ICL adjusts itself to a new shape without the need for active control loops, preventing the build-up of a high loading pressure. The viscoelastic property is generally considered a disadvantage due to loss of positional accuracy. However, a large number of potential biomaterial manipulation cases require the application of a differential mechanical stimulus (e.g., a gentle touch), whereas referencing to an absolute position may be of secondary importance.

**Figure 10:**
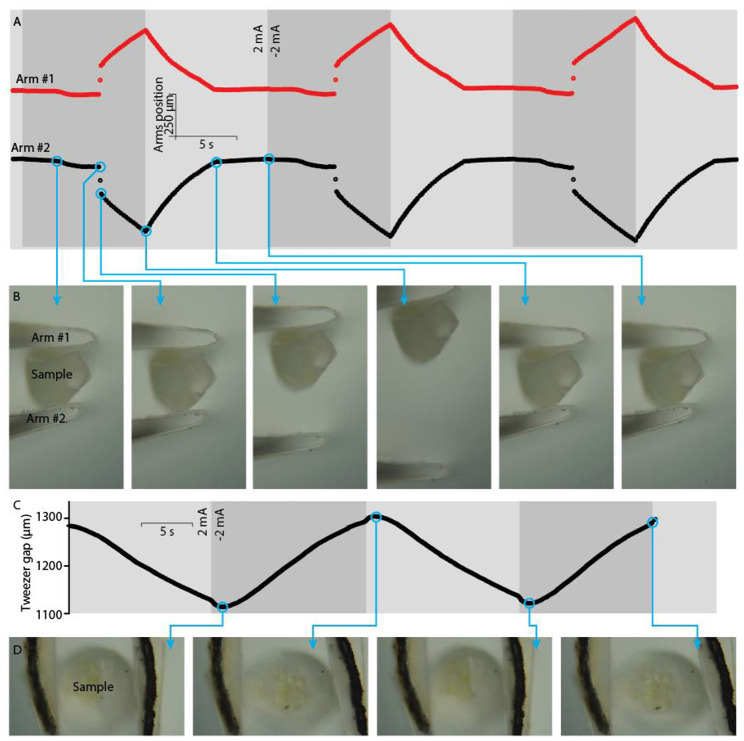
Manipulation of a soft biological sample, a capelin egg. **(A)** Positions of tweezer arm tips while repetitive clamping-releasing of a capelin egg, and **(B)** optical micrographs corresponding to the indicated time steps. **(C)** Position of tweezer arms while squeezing a capelin egg, and **(D)** the corresponding optical micrographs.

The intrinsically soft ICL manipulator is particularly well-suited for probing soft samples, offering the necessary safety margin and conforming to samples of non-uniform geometries. Figures 10A,B demonstrate the ICL-based active tweezers gripping a large cell, a single capelin egg. The ICL provides the intrinsic safety for such manipulation: the tweezers can grasp a highly delicate object and squeeze it with minimum risk of damage thanks to the compliant nature of the soft actuator.

The electroactive tweezer arms were used to manipulate a delicate capelin egg as follows. First, the sample was attached to the grips at zero charge; the gripped load adjusted itself in time so that the contact pressure approached zero. Then, a differential load was applied by injecting charge to the ICL to clamp the capelin egg with the tweezers. Figures 10C,D depict the mechanical squeezing of a capelin egg between the tweezer arms (see also Supplementary Video 2). The gap distance (Figure 10C) adjusted itself according to the sample size, and the compliant nature of the ICL material prevented the build-up of possible large stresses upon a possible (spontaneous) change in the sample geometry. The tweezer gap distance modulated by ~200 μm, indicating compliant and soft deformation applied to the capelin egg sample (Figure 10D).

### Manipulator Operation in Vacuum

**Figure 11:**
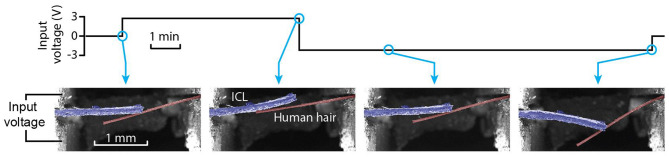
Image sequence of an ICL actuator (false-colored in blue) probing human hair (false-colored in brown) in a vacuum.

The ICL includes a liquid electrolyte essential to its working principle. This raises the question—are the ICL manipulators stable in the vacuum chamber of conventional SEM instruments? A simple biological sample (a human hair) was manipulated in the SEM vacuum chamber to prove that the ICL material can also be used to probe samples in the vacuum environment. Figure 11 shows the highly miniaturized setup of a soft ICL manipulator and a soft sample, accommodated in an extremely confined space in the SEM chamber (2 mm longest dimension). Moreover, as the ICL is conductive, it is not subjected to the charging effects, unlike the non-conductive hair (see also Supplementary Video 3). Indeed—charging effects were observed in case of the hair, expressed as the bending of the electron beam upon passing near the charged hair in Figure 11, whereas no deflection of the electron beam was noticed in case of the conductive ICL. As the driving voltages for the ICL material are orders-of-magnitude lower than the acceleration voltage of the electron beam used in SEM, also no interference by the actuating voltage to the electron beam was registered during the operation of an ICL that was fully exposed to the electron beam. This is a clear advantage over the electrostatically-driven actuators, such as comb drives and dielectric elastomers that rely on high actuation voltage levels (in kilovolts) that can interact and interfere with the electron beam if used in a similar configuration as the ICL probe introduced here.

### Simultaneous Electrical and Mechanical Probing

**Figure 12:**
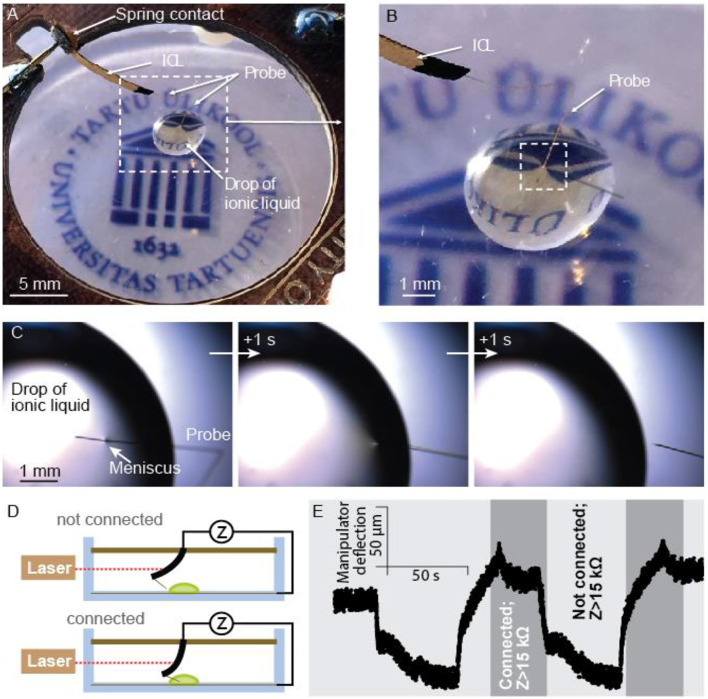
**(A)** The ICL manipulator probing a drop of liquid, and **(B)** closeup of the same. **(C)** A sequence of micrographs upon breaking the contact in-between the liquid sample and the probe. **(D)** Schematic description of the continuity measurement setup, and **(E)** probe's displacement at 5 mm from the contacts and formation and loss of electrical contact to the sample monitored by EIS (1 kHz, 5 mV_RMS_) between the integrated probe and a counter electrode (W/Au wire) on the bottom of the Petri dish.

The integration of conductive probes or sensors into the ICL opens up the possibility to characterize the mechanical and electrical properties of a sample (even simultaneously). As the current and voltage levels to drive an ICL are small, the interference from the actuation signal to the conductivity measurements is considered to be negligible. The possibility to measure the electrical properties of various samples was evaluated with the wire-carrying manipulator by probing a conductive sample—a drop of ionic liquid (Figures 12A,B). Figure 12C illustrates the probe and the droplet under a microscope. The surface tension force, as evidenced by the formation of a meniscus upon contact with the sample, did not significantly interfere with the actuation of the probe tip. The ICL-actuated probe tip was highly responsive under magnification, crossing the field-of-view in a few seconds, again showing that the ICL can fully cover the speed range relevant to most common micrographic techniques.

Moreover, the electrical properties of the assembly were tested using a simple continuity test. The actuation of the ICL manipulator (measured using a laser distance meter at 5 mm from the manipulator's base) reversibly created contact between the integrated probe and the liquid sample, as schematically shown in Figure 12D. The registered continuity data and the cantilever actuation is shown in Figure 12E. Combining the electrical measurement capabilities with the high-precision control introduced in more detail with active tweezers and the miniaturizability shown in the SEM probing section could be, for example, crucial for spatially dense manipulation scenarios of unknown and complicated samples.

## Conclusions

The ICLs have previously been demonstrated primarily for their actuation capability. We have explored how small changes in the manufacturing process can render these materials useful for prospective technologies of miniaturized manipulators. We have shown that ionic-liquid-based ICLs can be operated in air or in a vacuum, enabling probing of biological or other samples under an optical as well as a scanning electron microscope. The latter is enabled by the non-evaporative nature of ionic liquids and by the negligible interference from actuators to the electron optics.

The application-based design of ICL manipulators is an essential step ahead—instead of just characterizing the actuating material, we modified the ICL assembly procedure to construct practically relevant devices, i.e., a manipulator for interacting with soft (biological) samples. We explored four different manipulator configurations and demonstrated the integration of various tools—a simple cantilever probe, a needle probe, a capillary tube, and tweezer arms that are then steered by the ICL actuator.

The capillary tube integrated into the ICL's textile base demonstrates new application possibilities that take advantage of the uniform and widely distributed nature of textiles that have a well-defined structure in the mm-scale, but span in meters in two dimensions. The integration of fluidics in a self-morphing flexible textile platform is an essential step toward new health and care applications.

However, the biocompatibility of the actuators is of crucial importance when probing living tissues, cells, or other biological samples. ICL does offer actuation at relatively low potentials, thus providing a high safety margin in terms of electrical control. However, the choice of ICL constituent materials has, to date, been driven primarily by their impact on the composite actuation performance, consequently reaching to harmful substances (e.g., carbon nanotubes or some ionic liquids), and little research has so far been focused on the safety and biocompatibility of ICL materials. There have been attempts to encapsulate ICL materials with biocompatible passive layers. However, every passive addition to the active material reduces its maximum achievable deflection. Therefore, further development is suggested in the field of efficient encapsulation methods to enable safe probing of various samples in liquid, vacuum, or air without compromising the actuation performance to a large degree.

## Data Availability Statement

All datasets generated for this study are included in the article/Supplementary Material.

## Author Contributions

IM designed and analyzed the active tweezer, performed data analysis, participated in writing, and revising of the manuscript. PR did the comparative characterization of manipulator concepts, designed and analyzed the probe manipulator, performed data analysis, participated in writing, and revising of the manuscript. FKr designed, prepared, and analyzed the active pipette tip. FKa performed the vacuum manipulation study. UJ took part in the discussions and revised the manuscript. AA took part in the discussions.

### Conflict of Interest

The authors declare that the research was conducted in the absence of any commercial or financial relationships that could be construed as a potential conflict of interest.

## Supplementary Material

Supplementary Video 1 Demonstration of an active, ICL-driven pipette for positioning liquids.

Supplementary Video 2 Squeezing of a soft sample, a single capelin egg, using ICL tweezers.

Supplementary Video 3 *In situ* bending of a biological sample—a human hair—using an ICL manipulator in the vacuum of the SEM chamber.
